# Breast cancer in northern Peru: molecular subtypes and HER2 low

**DOI:** 10.17843/rpmesp.2024.411.13424

**Published:** 2024-03-25

**Authors:** Katherine Gómez-Rázuri, Milagros Abad-Licham, Juan Astigueta, Joan Moreno

**Affiliations:** 1 Regional Institute of Neoplastic Diseases of the North. La Libertad, Peru. Regional Institute of Neoplastic Diseases of the North La Libertad Peru; 2 School of Human Medicine, Universidad Privada Antenor Orrego. La Libertad, Peru. Universidad Privada Antenor Orrego School of Human Medicine Universidad Privada Antenor Orrego La Libertad Peru

**Keywords:** Breast Neoplasms, inmunohistochemistry, receptor ErbB-2, biomarkers tumor

## Abstract

This study aimed to understand the immunohistochemical profile of breast cancer and to identify the HER2 low subgroup in the northern macro-region of Peru. A cross-sectional study was conducted in 1176 patients from the Regional Institute of Neoplastic Diseases Northern Peru, from January 2016 to December 2023. We analyzed the data (age, histological type, grade and complementary results), with frequencies and percentages. The profile corresponded to: luminal B (45.6%); luminal A (24.7%); triple negative (18.2%); and HER2 positive non luminal (11.5%). In addition, 215 patients presented HER2 low (25.1% of those previously considered negative). This study provides evidence that the subtyping of breast cancer has changed, being luminal B the most frequent. It is essential to involve health policies to acquire targeted therapies considering HER2 low patients.

## INTRODUCTION

Breast cancer is a public health problem, being the most frequent malignant neoplasm in women worldwide (31%), and the second cause of female oncologic death in low- and middle-income countries (15%) ^1^. In Peru, its incidence has increased by 0.5% annually since mid-2000, and its prevalence is 135 per 100,000 inhabitants ^2^.

Breast cancer is characterized by its heterogeneity, both morphologically and in biological behavior, clinical course and prognosis. The current classification established by the World Health Organization (WHO) considers breast cancer as: no special type (NOS), which corresponds to 75%, and special types. However, this assessment showed gaps and limitations of clinical impact ^3^.

With the concept of personalized medicine, breast cancer reached a more precise diagnosis with molecular classification ^4^. At the beginning of the 21st century, Perou *et al*. carried out a study that evolved into four intrinsic molecular subtypes: luminal A, luminal B, non-luminal positive human epithelial growth factor receptor 2 (HER2) gene, and triple negative. In the latter, the basal type predominates (70-80%) which, from a biological perspective, could be considered a type of cancer by its own ^5^.

Due to the complexity and cost of gene profiling, we were able to simulate these results by immunohistochemical (IHC) study. The basic panel consists of: estrogen receptor (ER), progesterone receptor (PR), HER2 and Ki-67, whose criteria was las modified by the European Consensus of St. Gallen in 2013 ^6^. This intrinsic molecular subtyping is as follows: luminal A is ER positive and/or RP positive with HER2 negative and has low Ki-67 (less than 20%); luminal B is ER positive and/or RP positive, HER2 positive or negative, and has high Ki-67 (greater than or equal to 20%); HER2 positive non luminal is ER negative, RP negative, and HER2 positive; and triple negative is ER negative, RP negative, HER2 negative ^4^^,^^6^. The WHO reports that the frequency of these subtypes is as follows: luminal A (40-60%), followed by luminal B (20-30%), triple negative (basal type) (15-20%), and the HER2 positive non luminal subtype (10-20%) ^3^.

In Peru, four studies have described the profile of the molecular subtypes of breast cancer, three of them were developed in Lima and one in Arequipa. The first one, carried out between 2000 and 2002 by Vallejos *et al*., included 1198 patients and concluded that the predominant subtype was luminal A (49.3%), followed by triple negative (21.3%), HER2 positive non luminal (16.2%), and luminal B (13.2%) ^7^. Subsequently, Medina, in the years 2009 to 2012, in a series of 280 participants found the following rates: luminal A (37.5%), luminal B (31.4%), HER2 positive non luminal (16.4%), and triple negative (14.6%) ^8^. Between 2015 to 2017, Chachaima *et al.*, conducted a study in 259 patients and reported the following: luminal A (40.1%), luminal B (32.4%), triple negative (15.4%), and HER2 positive non luminal (11.9%) ^9^. Finally, Zavala *et al*., in 2022, analyzed hormone receptor (HR) and HER2 expression in 1943 breast cancer patients, finding the following: HR positive with HER2 negative (52.4%), HR positive with HER2 positive (18.7%), HR negative with HER2 positive (12.9%), and HR negative with HER2 negative (16.0%) ^10^.

HER2 is an important prognostic and predictive biomarker in breast cancer. It is currently classified as positive when it scores 3+ by immunohistochemistry, or scores 2+ with HER2 gene amplification by *in situ* hybridization (ISH). This represents a targeted anti-HER2 therapeutic opportunity. However, the dichotomy of this receptor (positive or negative) has been overturned with the introduction of new antibody-drug conjugates (ADCs) ^11^^-^^13^.

The IHC study of HER2 was designed to differentiate high levels of its expression (almost 2 million molecules per cell for 3+ score) from lower levels (20,000 to 500,000 molecules per cell for 0 to 2+ scores). The concept of “HER2 low” has emerged in recognition of this and of the intra-tumoral heterogeneity. ^11^^,^^12^. This was defined in the DESTINY-Breast04 study as tumors expressing an immunohistochemical score of 1+, or 2+ without gene amplification by ISH. The randomized, multicenter, open-label clinical trial involved 557 patients with unresectable or metastatic HER2 low breast cancer, who after receiving targeted therapy showed improvement in overall and progression-free survival. The Food and Drug Administration (FDA) approved Trastuzumab-deruxtecan (T-Dxd) therapy in this subgroup in August 2022 ^11^^,^^13^.

The aim of this study was to describe the immunohistochemical profile of breast cancer and to identify the subgroup of HER2 low patients in the northern macroregion of Peru.

**KEY MESSAGES**

Motivation for the study: Molecular classification of breast cancer allows the use of targeted treatments. Information on this profile in the northern macroregion of Peru is unknown. In addition, new therapies have appeared for a subgroup of patients.

Main findings: In this study, the most frequent molecular subtypes were: luminal B, luminal A, triple negative and non-luminal HER2. Also, 18.3% of patients had low HER2 expression.

Implications: Health policies should be aligned with scientific advances, to guarantee targeted therapies and to update the information in health manuals or protocols.

## THE STUDY

We conducted a descriptive, cross-sectional, retrospective study. Medical records and complementary studies were reviewed in patients with breast cancer from January 2016 to December 2023, attended at the Instituto Regional de Enfermedades Neoplásicas Norte (IREN Norte) in Peru. The inclusion criteria were to have histological diagnosis of breast cancer, complete immunohistochemistry study, and *in situ* hybridization (ISH) if accurate. Data were collected in Microsoft Excel 2016 and the variables of interest were: age, histologic type, histologic grade, immunohistochemistry and *in situ* hybridization results.

The histopathological study was carried out on core biopsies of primary breast tumors, fixed in buffered formalin and embedded in kerosene according to laboratory standards. The histological type was considered according to WHO as NOS, lobular or others. The histological grade was evaluated according to the Elston-Ellis modification of the Bloom Richardson system. The markers in the immunohistochemical study were: RE (window confirm anti-estrogen Clone SP1), RP (window confirm anti-progesterone Clone 1E2), HER2/neu (window confirm anti-Her-2/neu PATHWAY Clone 4B5), and Ki67 (window confirm anti Ki-67 30-9 Monoclonal). RE or RP was considered positive, according to the internationally agreed proportion and intensity score with an allred score greater than or equal to three points. Regarding HER2, we followed the 2018 recommendations of the American Society of Clinical Oncology (ASCO) and the College of American Pathologists (CAP), ratified at the date of the study. This immunohistochemical interpretation yields the following results: 3+ (positive), 2+ (equivocal), 1+ (negative) and 0 (negative). Where, if “equivocal”, the receptor study was continued by in situ hybridization (dual window ISH DNA Probe Cocktail Assay) with positive or negative result according to the HER2/CEP17 ratio (chromosome 17 centromeric probe). The cut-off point of 20% was considered for Ki67 according to the St. Gallen 2013 consensus discussion (3,14,15).

For the definition of breast cancer subtype we considered: luminal A (ER positive and/or PR positive with HER2 negative and Ki-67 less than 20%); luminal B (ER positive and/or PR positive, HER2 positive or negative, and Ki-67 greater than or equal to 20%); HER2 positive non luminal (ER negative, PR negative, and HER2 positive); and triple negative (ER negative, PR negative, HER2 negative). We defined cases of “HER2 low” patients as those with immunohistochemical results 1+, or 2+ with negative ISH ^4^^,^^6^^,^^13^.

We analyzed the variables of interest including the immunohistochemical profile in breast cancer, as well as the “HER low” cases. Frequencies and percentages are presented in tables.

This study was approved by the ethics committee of the Instituto Regional de Enfermedades Neoplásicas del Norte. The code was: 024-2023-IREN NORTE-CIEI.

## FINDINGS

Of 1202 patients with breast cancer diagnosed at IREN Norte, 26 were excluded due to incomplete immunohistochemical study, resulting in a sample of 1176 patients.

The most frequent age at diagnosis was 52 years. The distribution according to the histologic type was: no special type (NOS) in 852 (72.5%); lobular in 280 (23.8%); and others in 44 (3.7%) patients. Regarding the histological grade: 18 patients were G1 (1.5%); 723 patients, G2 (61.5%); and 435 patients G3 (37.0%) (Table 1).

**Table 1:** Age and histological characteristics according to molecular subtype of breast cancer in patients from the Regional Institute of Neoplastic Diseases of the North, Peru 2016 to 2023.

		Luminal A		Luminal B		Luminal B HER2		HER2		Triple negative		Total	
		cases	%	cases	%	cases	%	cases	%	cases	%	cases	%
Age													
	20-39	28	15.6	54	30.0	22	12.2	27	15.0	49	27.2	180	15.3
	40-59	146	22.4	194	29.8	120	18.4	74	11.3	118	18.1	652	55.4
	60-79	103	32.7	97	30.8	41	13.0	31	9.8	43	13.7	315	26.8
	80 or more	14	48.3	6	20.7	3	10.3	2	6.9	4	13.8	29	2.5
Histological type													
	NOS	182	21.4	255	29.9	133	15.6	105	12.3	177	20.8	852	72.5
	Lobular	88	31.4	91	32.5	47	16.8	25	8.9	29	10.4	280	23.8
	Other	21	47.7	5	11.4	6	13.6	4	9.1	8	18.2	44	3.7
Histological grade													
	G1	11	61.1	3	16.7	1	5.6	1	5.6	2	11.1	18	1.5
	G2	228	31.5	221	30.6	120	16.6	81	11.2	73	10.1	723	61.5
	G3	52	12.0	127	29.2	65	14.9	52	12.0	139	32.0	435	37.0

HER2: human epidermal growth factor receptor 2, NOS: no special type, G1: histologic grade 1, G2: histologic grade 2, G3: histologic grade 3

Regarding the molecular profile, the most frequent subtype was luminal B in 537 patients (45.6%), of which 186 expressed HER2, followed by luminal A with 291 (24.7%), triple negative in 214 (18.2%) and finally HER2 positive non-luminal in 134 (11.5%). When evaluating the distribution by year, we found that in 2016, 50.0% were luminal A cases, decreasing to less than half in the year 2023 with 21.0%. The opposite occurred with the luminal B subtype, which in 2016 was 21.4%, and in 2023 represented 54.8%. The subtypes: triple negative and HER2 positive non luminal, have shown little variation over the years, with a slight increase in the latter from 8.9% (2016) to 10.8% (2023) (Table 2).

**Table 2:** Molecular subtyping of breast cancer by year at the Regional Institute of Neoplastic Diseases of the North, Peru 2016 to 2023.

Year	Luminal A		Luminal B		Luminal B HER2		HER2		Triple negative		Total by year	
	Cases	%	Cases	%	Cases	%	Cases	%	Cases	%	Cases	%
2016	28	50.0	5	8.9	7	12.5	5	8.9	11	19.6	56	4.8
2017	33	37.9	14	16.1	17	19.5	9	10.3	14	16.1	87	7.4
2018	50	32.9	27	17.8	23	15.1	26	17.1	26	17.1	152	13.0
2019	52	29.9	52	29.9	25	14.4	16	9.2	29	16.7	174	14.8
2020	26	16.1	53	32.9	26	16.1	13	8.1	43	26.7	161	13.7
2021	23	14.2	70	43.2	20	12.3	22	13.6	27	16.7	162	13.8
2022	38	20.1	64	33.9	27	14.3	22	11.6	38	20.1	189	16.0
2023	41	21.0	66	33.8	41	21.0	21	10.8	26	13.3	195	16.5
Total	291	24.7	351	29.8	186	15.8	134	11.5	214	18.2	1,176	100.0

HER2: human epidermal growth factor receptor 2.

**Figure 1:**
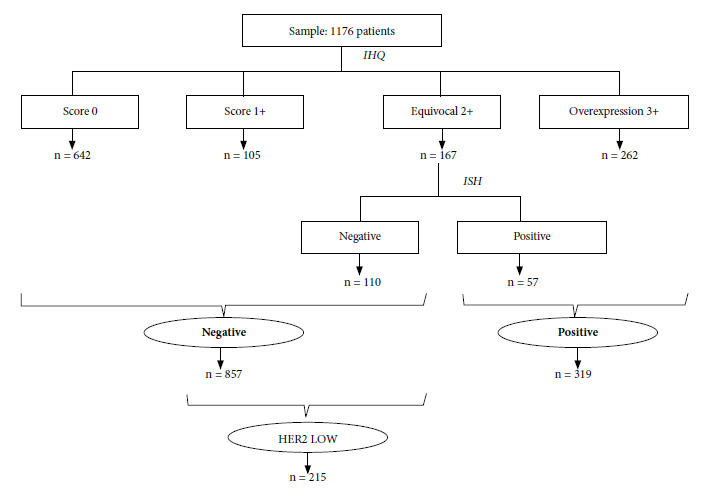
HER2 status according to complementary studies at the Instituto Regional de Enfermedades Neoplásica del Norte, Peru 2016 to 2023.

We obtained the following HER2 expression and interpretation results, the most frequent mode was negative (score 0) in 642 patients, negative (1+) in 105 patients, equivocal/doubtful (2+) in 167 patients, and positive (3+) in 262 patients. After the ISH study in equivocal cases, patients expressed dichotomous results: positive (319 patients) and negative (857 patients). The “HER2 low” case definition was applied in the latter group, with 215 patients (18.3% of the total sample and 25.1% of the negative subgroup) meeting these criteria (Figure 1).

Of the latter group, 131 patients had intermediate histologic grade (G2) and 163 patients were histologic type NOS. Regarding the molecular subtype of the HER2 low patients, 78 patients were luminal A, 107 luminal B, and 30 triple negative (Table 3).

**Table 3:** Characteristics of patients with “HER2 low” breast cancer at the Regional Institute of Neoplastic Diseases of the North, Peru 2016 to 2023.

		Luminal A		Luminal B		Triple negative		Total	
		Cases	%	Cases	%	Cases	%	Cases	%
Age									
	20-39	9	25.7	19	54.3	7	20.0	35	16.3
	40-59	42	35.9	61	52.1	14	12.0	117	54.4
	60-79	23	41.1	26	46.4	7	12.5	56	26.0
	80 or more	4	57.1	1	14.3	2	28.6	7	3.3
Histological type									
	NOS	53	32.5	87	53.4	23	14.1	163	75.8
	Lobular	20	43.5	20	43.5	6	13.0	46	21.4
	Other	5	83.3	0	0.0	1	16.7	6	2.8
Histological grade									
	G1	1	33.3	2	66.7	0	0.0	3	1.4
	G2	58	44.3	62	47.3	11	8.4	131	61.0
	G3	19	23.5	43	53.1	19	23.5	81	37.6
	Total	78	36.2	107	49.8	30	14.0	215	100.0

NOS: no special type, G1: histologic grade 1, G2: histologic grade 2, G3: histologic grade 3.

## DISCUSSION

In this study we analyzed the distribution of molecular subtypes of breast cancer, as well as data with prognostic value and those considered “HER2 low”. The most affected population group was those between 40 and 59 years of age. The American Cancer Society recommends that people aged 45 to 54 years should undergo annual mammography screening. In addition, the most frequent breast cancer in young women aged 20 to 40 years was triple negative, which is known to be more aggressive. Breast cancer in young women represents a significant burden for low- and middle-income countries, with more than 20% of cases occurring in women under 45 years of age ^1^^,^^2^^,^^15^.

Similar to our study, the highest proportion of high-grade breast cancer (G3) corresponded to the triple negative and HER2 non luminal subtypes in India, United States, Iran, Egypt, Morocco, Japan and Korea. On the other hand, most low-grade tumors (G1) were luminal A type ^3^^,^^9^^,^^16^. The most frequent histological grade was G2 (61.5%), followed by G3 (37.0%) and G1 (1.5%). The same order of frequencies was reported by Firdaus *et al.*, (G2 - 62.1%, G3 - 26.8% and G1 - 9.1%) ^17^.

The molecular profile in the 1176 patients from our study differs from the international and national literature. According to WHO, the most frequent subtype is luminal A with 40 to 60%, and the national studies reviewed coincide with this information. However, in our population it was in second place with only 24.7%. On the other hand, luminal B breast cancer was the most frequent subtype with 45.6% compared to 20.0% reported by the international literature and up to 32.0% in national studies ^7^^,^^8^^,^^9^.

In addition, the analysis by year showed that the frequency varied. From 2016 to 2023 there was an inversion of percentages of luminal A and luminal B breast cancer, in favor of the latter. This is a worrying situation since luminal A tumors have better prognosis, longer survival and lower recurrence among all subtypes ^3^^,^^8^. This may be due, in theory, to two circumstances, that there really is an increase in luminal subtype B, or that diagnostic tests have improved its recognition. In this article, we believe that both situations are feasible.

The first scenario could be caused by new mutations as some studies suggest that luminal B and other hormone-resistant breast cancer subtypes evolve from luminal A cancer. Estrogen being a dynamic regulator of several factors, loss of estrogen function and increased growth factor receptor (GFR) may occur during breast cancer progression to a hormone-resistant state ^18^.

In the second scenario, the role of the pre-analytical phase was of vital importance, standardizing the optimal processes for reliable immunohistochemical and molecular results. Thus, it became known that prolonged ischemia by late fixation decreases receptor expression (PR begins to decrease after one hour, ER after two hours, and after eight hours both expressions become completely negative) and that the fixation time for optimal receptor expression is a minimum of six to eight hours, while the maximum fixation time without a change in ER, PR, HER2 and ki67 expression is 72 hours. This led to the development of international guidelines and recommendations that regulated the time of ischemia, fixation, processing and reading, in order to limit the pre-analytical variables achieving a more accurate immunohistochemistry ^19^.

On the other hand, we evidenced a similar frequency of the most aggressive subtypes, triple negative (18.2%) and HER2 positive non luminal (11.5%), compared to the most current national study with 16.0% for triple negative (negative HR with HER2 negative) and 12.9% for HER2 positive non luminal (negative HR with HER2 positive) ^10^.

When HER2 status was assessed, we found that 215 patients met the criteria for the “low” subgroup. However, there is no national data to compare this finding. Internationally, it is reported that almost half of HER2-negative breast cancers show some degree of expression (“HER2 low”), but in our study it represented only 25.1% ^12^^,^^13^^,^^20^^,^^21^.

The molecular classification has not changed and HER2 low cannot be considered a molecular subtype, but it is important to recognize these patients since in the DESTINY-Breast04 clinical trial, trastuzumab deruxtecan (T-DXd) demonstrated that even fewer HER2 receptors on cancer cells may be sufficient for clinical benefit ^20^^,^^21^. Evaluation of this subgroup, especially in those triple-negative patients, is vital as it will allow targeted therapies, new clinical trials and the development of more accurate techniques to assess HER2 status ^22^.

Our study had some limitations such as the lack of national or regional statistics on HER2 low patients for comparison of results, the absence of data in the medica records on the current status of the patients, making it difficult to analyze the survival of a large part of the sample, and the absence of genetic tests that could enrich the results.

The subtyping profile of breast cancer in patients diagnosed at the Northern Regional Institute of Neoplastic Diseases during 2016 to 2023 differs from previous studies, showing a notable increase in luminal B subtype breast cancer at the expense of a decrease in luminal A subtype. Likewise, we found that the HER2 low subgroup represents a quarter of those patients previously considered without expression of these receptors, opening a therapeutic opportunity for them. This implies that public health policies must be involved and change according to the context. There must be a permanent contribution to research and more effective early detection programs, besides, specific treatments must be acquired, trained human resources must guarantee, and alliances for personalized medicine must be created.
