# Supplementary material for: Breast cancer in northern Peru: molecular subtypes and HER2 low
Source: Rev Peru Med Exp Salud Publica. 2024 Mar 25;41(1):62–8. doi: 10.17843/rpmesp.2024.411.13424 (PMC11149756; doi:10.17843/rpmesp.2024.411.13424)
Supplement: Translation

# Translation: Cáncer de mama en el norte del Perú: subtipos moleculares y HER2 low

**Authors:** Katherine Gómez-Rázuri, Milagros Abad-Licham, Juan Astigueta, Joan Moreno

**Affiliations:** 1 Instituto Regional de Enfermedades Neoplásicas del Norte. La Libertad, Perú.; 2 Escuela de Medicina Humana, Universidad Privada Antenor Orrego. La Libertad, Perú.

**Keywords:** Neoplasias de la mama, inmunohistoquímica, receptor ErbB-2, biomarcadores de tumor

## Abstract

El objetivo del estudio fue conocer el perfil inmunohistoquímico del cáncer de mama e identificar el subgrupo HER2 low en la macrorregión norte del Perú. Se realizó un estudio transversal con una muestra de 1176 pacientes atendidos en el Instituto Regional de Enfermedades Neoplásicas Norte del Perú desde enero de 2016 a diciembre de 2023. Los datos recolectados (edad, tipo histológico, grado y resultados complementarios), se analizaron con frecuencias y porcentajes. El perfil correspondió a: luminal B (45,6%); luminal A (24,7%); triple negativo (18,2%); y HER2 positivo no luminal (11,5%). Además, HER2 low fueron 215 pacientes (25,1% de los considerados previamente negativos). Este estudio proporciona evidencia que la subtipificación de cáncer de mama ha cambiado, siendo luminal B más frecuente, y es esencial involucrar a políticas de salud para adquirir terapias dirigidas considerando a pacientes HER2 low.

## INTRODUCCIÓN

El cáncer de mama es un problema de salud pública, siendo la neoplasia maligna más frecuente en mujeres a nivel mundial (31%), y la segunda causa de muerte oncológica femenina en países con ingresos bajos y medios (15%) ^1^. En Perú, su incidencia aumenta en 0,5% anual desde mediados del año 2000, y su prevalencia es 135 por 100 000 habitantes ^2^.

El cáncer de mama se caracteriza por su heterogeneidad, tanto morfológica, como en comportamiento biológico, curso clínico y pronóstico. La clasificación vigente establecida por la Organización Mundial de la Salud (OMS) considera al cáncer de mamá como: sin tipo especial (NOS), que corresponden al 75%, y tipos especiales. Sin embargo, esta valoración mostró carencias y limitaciones de impacto clínico ^3^.

Con el concepto de medicina personalizada, el cáncer de mama llegó a un diagnóstico más preciso con la clasificación molecular ^4^. Perou *et al*, realizaron a inicios del siglo XXI, el estudio que evolucionó en cuatro subtipos moleculares intrínsecos: luminal A, luminal B, gen del receptor 2 del factor de crecimiento epitelial humano (HER2) positivo no luminal, y triple negativo. En este último, predomina el tipo basal (70-80%) que, desde una perspectiva biológica, podría ser considerado un tipo de cáncer por sí mismo ^5^.

Por la complejidad y costos de los perfiles génicos, se logró simular estos resultados mediante el estudio inmunohistoquímico (IHC). El panel básico consta de: receptor de estrógenos (RE), receptor de progesterona (RP), HER2 y Ki-67, cuyos criterios tuvieron una última modificación en el Consenso Europeo de St. Gallen en el año 2013 ^6^. Esta subtipificación molecular intrínseca es la siguiente: luminal A presenta RE positivo y/o RP positivo con HER2 negativo y Ki-67 bajo (menor a 20%); luminal B, RE positivo y/o RP positivo, HER2 positivo o negativo, y Ki-67 alto (mayor o igual a 20%); HER2 positivo no luminal, RE negativo, RP negativo, y HER2 positivo; y triple negativo, RE negativo, RP negativo, HER2 negativo ^4^^,^^6^. La OMS refiere que la frecuencia de estos subtipos es a predominio de luminal A (40-60%), seguido de luminal B (20-30%), triple negativo (tipo basal) (15-20%), y el subtipo HER2 positivo no luminal (10-20%) ^3^.

En Perú, hay cuatro estudios que describen el perfil de los subtipos moleculares de cáncer de mama, tres de ellos desarrollados en Lima y uno en Arequipa. El primero, realizado entre 2000 y 2002 por Vallejos *et al*, estudió 1198 pacientes y concluyó como subtipo predominante luminal A (49,3%), seguido por triple negativo (21,3%), HER2 positivo no luminal (16,2%), y luminal B (13,2%) ^7^. Posteriormente, Medina en los años 2009 a 2012, en una serie de 280 encontró: luminal A (37,5%), luminal B (31,4%), HER2 positivo no luminal (16,4%), y triple negativo (14,6%) ^8^. Entre los años 2015 a 2017, Chachaima *et al*, de 259 pacientes publicaron los siguientes resultados: luminal A (40,1%), luminal B (32,4%), triple negativo (15,4%), y HER2 positivo no luminal (11,9%) ^9^. Finalmente, Zavala *et al*, en el año 2022, analizaron la expresión de los receptores hormonales (HR) y HER2 en 1943 pacientes con cáncer de mama, encontrando lo siguiente: HR positivo con HER2 negativo (52,4%), HR positivo con HER2 positivo (18,7%), HR negativo con HER2 positivo (12,9%), y HR negativo con HER2 negativo (16,0%) ^10^. 

El HER2 es un biomarcador pronóstico y predictivo importante en cáncer de mama. Actualmente, se clasifica como positivo cuando obtiene una puntuación de 3+ mediante inmunohistoquímica, o una puntuación de 2+ con amplificación del gen HER2 mediante hibridación *in situ* (ISH). Ello significa una oportunidad terapéutica dirigida anti-HER2. Sin embargo, esta dicotomía del receptor (positivo o negativo) ha sido derribada con la introducción de nuevos conjugados de fármaco-anticuerpo (ADC) ^11^^-^^13^.

El estudio IHC de HER2 se diseñó para diferenciar niveles altos de su expresión (casi 2 millones de moléculas por célula para puntuación 3+) de niveles más bajos (20 000 a 500 000 moléculas por células para puntuaciones de 0 a 2+). Conscientes de esto y de la heterogeneidad intratumoral, aparece el concepto “HER2 low” ^11^^,^^12^. Éste fue definido en el estudio DESTINY-Breast04 como aquellos tumores que expresan una puntuación inmunohistoquímica 1+, o 2+ sin amplificación del gen por ISH. En el ensayo clínico aleatorizado, multicéntrico y abierto participaron 557 pacientes con cáncer de mama HER2 low irresecable o metastásico, quienes luego de recibir terapia dirigida mostraron mejora en la sobrevida global y libre de progresión de enfermedad. La Food and Drug Administration (FDA) aprobó en agosto del 2022 la terapia con Trastuzumab-deruxtecan (T-Dxd) en este subgrupo ^11^^,^^13^.

El objetivo del presente estudio fue conocer el perfil inmunohistoquímico del cáncer de mama e identificar el subgrupo de pacientes HER2 low en la macrorregión norte del Perú.

**MENSAJE CLAVE**

Motivación para realizar el estudio: La clasificación molecular del cáncer de mama permite utilizar tratamientos dirigidos. La información de este perfil en la macrorregión norte del Perú es desconocida. Además, han aparecido nuevas terapias para un subgrupo de pacientes.

Principales hallazgos: En este estudio, los subtipos moleculares por orden de frecuencia fueron: luminal B, luminal A, triple negativo y HER2 no luminal. Asimismo, 18,3% de pacientes tuvieron expresión HER2 low.

Implicancias: Las políticas de salud deben corresponderse con los avances científicos, para garantizar terapias dirigidas y actualizar la información de los manuales o protocolos de salud.

## EL ESTUDIO

Se realizó un estudio descriptivo, transversal y retrospectivo. Se revisaron historias clínicas y estudios complementarios en pacientes con cáncer de mama desde enero de 2016 a diciembre de 2023, atendidos en el Instituto Regional de Enfermedades Neoplásicas Norte (IREN Norte) de Perú. Los criterios de inclusión fueron contar con diagnóstico histológico de cáncer de mama, estudio de inmunohistoquímica completo, e hibridación *in situ* (ISH) de ser preciso. Los datos se recolectaron en Microsoft Excel 2016, siendo las variables de interés: edad, tipo histológico, grado histológico, resultados de inmunohistoquímica e hibridación *in situ*.

El estudio histopatológico fue en biopsias *core* de tumores primarios de mama, fijados en formol tamponado e incluidos en parafina según estándares del laboratorio. El tipo histológico fue considerado según la OMS como NOS, lobulillar u otros. El grado histológico se evaluó según modificación de Elston-Ellis del sistema Bloom Richardson. En el estudio inmunohistoquímico los marcadores fueron: RE (ventana *confirm anti-estrogen Clone* SP1), RP (ventana *confirm anti-progesterone Clone* 1E2), HER2/neu (ventana PATHWAY anti-Her-2/neu Clone 4B5), y Ki67 (*ventana confirm* anti Ki-67 30-9 Monoclonal). Considerándose positivo RE o RP, según puntaje de proporción e intensidad consensuado internacionalmente con un *allred score* mayor o igual a tres puntos. Para HER2, se siguió las recomendaciones de la Sociedad Americana de Oncología Clínica (ASCO) y el Colegio de Patólogos Americanos (CAP) del año 2018, ratificadas a la fecha del estudio. Esta interpretación inmunohistoquímica confiere resultados: 3+ (positivo), 2+ (equívoco), 1+ (negativo) y 0 (negativo). Donde, de ser “equívoco”, prosiguió el estudio del receptor mediante hibridación *in situ* (ventana dual ISH DNA *Probe Cocktail Assay*) con resultado positivo o negativo según relación HER2/CEP17 (sonda centromérica del cromosoma 17). Para Ki67 se consideró el punto de corte 20% según discusión de consenso St. Gallen 2013 ^3^^,^^14^^,^^15^.

Para la definición del subtipo de cáncer de mama se consideró: luminal A (RE positivo y/o RP positivo con HER2 negativo y Ki-67 menor a 20%); luminal B (RE positivo y/o RP positivo, HER2 positivo o negativo, y Ki-67 mayor o igual a 20%); HER2 positivo no luminal (RE negativo, RP negativo, y HER2 positivo); y triple negativo (RE negativo, RP negativo, HER2 negativo). Y para definir los casos de pacientes “HER2 low” se consideró los resultados inmunohistoquímicos 1+, o 2+ con ISH negativo ^4^^,^^6^^,^^13^.

Se realizó el análisis de las variables de interés incluyendo el perfil inmunohistoquímico en cáncer de mama, así como los casos “HER low”. Se hallaron las frecuencias y porcentajes, las cuales se presentan en tablas.

Se obtuvo la aprobación del comité de ética del Instituto Regional de Enfermedades Neoplásicas del Norte. El código fue: 024-2023-IREN NORTE-CIEI.

## HALLAZGOS

De 1202 pacientes con cáncer de mama diagnosticados en el IREN Norte, se excluyeron 26 debido a estudio inmunohistoquímico incompleto, dejando una muestra de 1176 pacientes.

La edad más frecuente al diagnóstico fue 52 años. Según el tipo histológico la distribución fue: ningún tipo especial (NOS) en 852 pacientes (72,5%); lobulillar, 280 (23,8%); y otros, 44 (3,7%). En relación con el grado histológico: 18 pacientes fueron G1 (1,5%); 723 pacientes, G2 (61,5%); y 435 pacientes G3 (37,0%) (Tabla 1).

**Tabla 1:** Edad y características histológicas según subtipo molecular de cáncer de mama en pacientes atendidas en el Instituto Regional de Enfermedades Neoplásica del Norte, Perú 2016 a 2023.

		Luminal A		Luminal B		Luminal B HER2		HER2		Triple negativo		Total	
		casos	%	casos	%	casos	%	casos	%	casos	%	casos	%
Edad													
	20-39	28	15,6	54	30,0	22	12,2	27	15,0	49	27,2	180	15,3
	40-59	146	22,4	194	29,8	120	18,4	74	11,3	118	18,1	652	55,4
	60-79	103	32,7	97	30,8	41	13,0	31	9,8	43	13,7	315	26,8
	80 o más	14	48,3	6	20,7	3	10,3	2	6,9	4	13,8	29	2,5
Tipo histológico													
	NOS	182	21,4	255	29,9	133	15,6	105	12,3	177	20,8	852	72,5
	Lobulillar	88	31,4	91	32,5	47	16,8	25	8,9	29	10,4	280	23,8
	Otros	21	47,7	5	11,4	6	13,6	4	9,1	8	18,2	44	3,7
Grado histológico													
	G1	11	61,1	3	16,7	1	5,6	1	5,6	2	11,1	18	1,5
	G2	228	31,5	221	30,6	120	16,6	81	11,2	73	10,1	723	61,5
	G3	52	12,0	127	29,2	65	14,9	52	12,0	139	32,0	435	37,0

HER2: receptor 2 del factor de crecimiento epidérmico humano, NOS: sin tipo especial, G1: grado histológico 1, G2: grado histológico 2, G3: grado histológico 3

Respecto al perfil molecular, el subtipo más frecuente fue luminal B en 537 pacientes (45,6%), de los cuales 186 expresaban HER2, seguido por luminal A con 291 (24,7%), triple negativo en 214 (18,2%) y finalmente HER2 positivo no luminal en 134 (11,5%). Al evaluar la distribución por año se encontró que en el 2016 el 50,0% de los casos correspondían a luminal A, disminuyendo a menos de la mitad en el año 2023 con 21,0%. Caso contrario ocurrió con el subtipo luminal B, que el 2016 era 21,4%, y el 2023 representa el 54,8%. Los subtipos: triple negativo y HER2 positivo no luminal, han mostrado poca variación a lo largo de los años, con un leve incremento de este último de 8,9% (2016) a 10,8% (2023) (Tabla 2).

**Tabla 2:** Subtipificación molecular de cáncer de mama por año en el Instituto Regional de Enfermedades Neoplásica del Norte, Perú 2016 a 2023.

Año	Luminal A		Luminal B		Luminal B HER2		HER2		Triple negativo		Total por año	
	Casos	%	Casos	%	Casos	%	Casos	%	Casos	%	Casos	%
2016	28	50,0	5	8,9	7	12,5	5	8,9	11	19,6	56	4,8
2017	33	37,9	14	16,1	17	19,5	9	10,3	14	16,1	87	7,4
2018	50	32,9	27	17,8	23	15,1	26	17,1	26	17,1	152	13,0
2019	52	29,9	52	29,9	25	14,4	16	9,2	29	16,7	174	14,8
2020	26	16,1	53	32,9	26	16,1	13	8,1	43	26,7	161	13,7
2021	23	14,2	70	43,2	20	12,3	22	13,6	27	16,7	162	13,8
2022	38	20,1	64	33,9	27	14,3	22	11,6	38	20,1	189	16,0
2023	41	21,0	66	33,8	41	21,0	21	10,8	26	13,3	195	16,5
Total	291	24,7	351	29,8	186	15,8	134	11,5	214	18,2	1,176	100,0

HER2: receptor 2 del factor de crecimiento epidérmico humano.

Sobre el estado de la expresión e interpretación de HER2, del modo estándar se obtuvo: negativo (puntuación 0) a 642 pacientes, negativo (1+) a 105 pacientes, equívoco/dudoso (2+) a 167 pacientes, y positivo (3+) a 262 pacientes. Después del estudio ISH en los casos dudosos, el resultado total de los pacientes expresado de manera dicotómica fue: positivo (319 pacientes) y negativo (857 pacientes). La definición de casos “HER2 low” se aplicó en este último grupo, hallándose 215 pacientes (18,3% del total de la muestra y 25,1% del subgrupo negativo) que cumplían con estos criterios (Figura 1).

**Figura 1:** Estado del HER2 según estudios complementarios en el en el Instituto Regional de Enfermedades Neoplásica del Norte, Perú 2016 a 2023.

De este último grupo, 131 pacientes tuvieron grado histológico intermedio (G2) y 163 pacientes fueron tipo histológico NOS. Respecto al subtipo molecular de los pacientes HER2 low, 78 pacientes fueron luminal A, 107 luminal B, y 30 triple negativo (Tabla 3).

**Tabla 3:** Características de los pacientes con cáncer de mama “HER2 low” en el Instituto Regional de Enfermedades Neoplásica del Norte, Perú 2016 a 2023.

		Luminal A		Luminal B		Triple negativo		Total	
		Casos	%	Casos	%	Casos	%	Casos	%
Edad									
	20-39	9	25,7	19	54,3	7	20,0	35	16,3
	40-59	42	35,9	61	52,1	14	12,0	117	54,4
	60-79	23	41,1	26	46,4	7	12,5	56	26,0
	80 o más	4	57,1	1	14,3	2	28,6	7	3,3
Tipo histológico									
	NOS	53	32,5	87	53,4	23	14,1	163	75,8
	Lobulillar	20	43,5	20	43,5	6	13,0	46	21,4
	Otros	5	83,3	0	0,0	1	16,7	6	2,8
Grado histológico									
	G1	1	33,3	2	66,7	0	0,0	3	1,4
	G2	58	44,3	62	47,3	11	8,4	131	61,0
	G3	19	23,5	43	53,1	19	23,5	81	37,6
	Total	78	36,2	107	49,8	30	14,0	215	100,0

* NOS: sin tipo especial, G1: grado histológico 1, G2: grado histológico 2, G3: grado histológico 3.

## DISCUSIÓN

En el presente estudio hemos analizado la distribución de los subtipos moleculares de cáncer de mama, así como datos con valor pronóstico y aquellos considerados “HER2 low”. El grupo poblacional más afectado fue el comprendido entre 40 a 59 años. La Sociedad Americana contra el Cáncer recomienda que las personas de 45 a 54 años deben someterse a una mamografía anual como tamizaje. Además, el cáncer de mama más frecuente en mujeres jóvenes de 20 a 40 años fue triple negativo el cual se conoce más agresivo. El cáncer de mama en mujeres jóvenes representa una carga significativa para los países con ingresos bajos y medios, ya que más del 20% de los casos se producen en mujeres de menores de 45 años ^1^^,^^2^^,^^15^.

De forma semejante al presente estudio, la mayor proporción de cáncer de mama de alto grado (G3) correspondió a los subtipos triple negativo y HER2 no luminal tal como en India, Estados Unidos, Irán, Egipto, Marruecos, Japón y Corea. Por otro lado, la mayor parte de los tumores de bajo grado (G1) fueron luminal A ^3^^,^^9^^,^^16^. El grado histológico más frecuente fue G2 (61,5%), seguido de G3 (37,0%) y G1 (1,5%). El mismo orden de frecuencia se observó en el estudio de Firdaus *et al*, (G2 - 62,1%, G3 - 26,8% y G1 - 9,1%) ^17^.

El perfil molecular hallado en los 1176 pacientes del presente estudio difiere de la bibliografía internacional y nacional. Siendo para la OMS el subtipo más frecuente luminal A con 40 a 60%, y los estudios nacionales revisados coinciden con esta información. Sin embargo, en nuestra población ocupa el segundo lugar con apenas 24,7%. Por otro lado, el cáncer de mama luminal B fue el subtipo más frecuente con 45,6% frente al 20,0% referido por la literatura internacional y hasta el 32,0% en estudios nacionales ^7^^,^^8^^,^^9^.

Además, el análisis por año mostró que la frecuencia presentó variaciones. Desde el año 2016 hasta 2023 hubo una inversión de porcentajes de cáncer de mama luminal A y luminal B, a favor del segundo. Esta es una situación preocupante ya que los tumores luminales A presentan un mejor pronóstico, mayor supervivencia y menor recurrencia entre todos los subtipos ^3^^,^^8^. Esto puede deberse, en teoría, a dos circunstancias, que realmente exista un incremento del subtipo luminal B, o que las pruebas diagnósticas hayan mejorado su reconocimiento. En el presente artículo, creemos que ambas situaciones son factibles.

En el primer escenario, las causas podrían ser nuevas mutaciones ya que existen estudios que sugieren que los subtipos de cáncer de mama luminal B y otros resistentes a hormonas evolucionan a partir de un cáncer luminal A. Al ser el estrógeno regulador dinámico de varios factores, la pérdida de su función y el aumento del receptor de factor de crecimiento (TFG) pueden ocurrir durante la progresión del cáncer de mama a un estado de resistencia hormonal ^18^.

En el segundo escenario, el rol de la fase preanalítica ocupó vital importancia, estandarizándose los procesos óptimos para resultados inmunohistoquímicos y moleculares fiables. Así, se conoció que la isquemia prolongada por fijación tardía disminuye la expresión de los receptores (RP comienza a disminuir tras una hora, RE después de dos horas, y tras ocho horas ambas expresiones se vuelven completamente negativas) y que el tiempo de fijación para la expresión óptima de los receptores es mínimo de seis a ocho horas, mientras que el tiempo de fijación máximo sin un cambio en la expresión de ER, PR, HER2 y ki67 es de 72 horas. Esto llevó a la elaboración de guías y recomendaciones internacionales que regularon el tiempo de isquemia, fijación, procesamiento y lectura, para limitar las variables preanalíticas logrando una inmunohistoquímica más exacta ^19^.

Por otro lado, se evidenció una frecuencia similar de los subtipos más agresivos, triple negativo (18,2%) y HER2 positivo no luminal (11,5%), frente al estudio nacional más actual con 16,0% para el triple negativo (HR negativo con HER2 negativo) y 12,9% para HER2 positivo no luminal (HR negativo con HER2 positivo) ^(^^10^.

Al evaluar el estado del HER2, 215 pacientes cumplían con los criterios referidos para el subgrupo “low”. Sin embargo, no existe data a nivel nacional para comparar este hallazgo. Internacionalmente, se indica que casi la mitad de los cánceres de mama HER2 negativos muestran algún grado de expresión (“HER2 low”), pero en el estudio representó solo el 25,1% ^12^^,^^13^^,^^20^^,^^21^.

La clasificación molecular no ha cambiado y HER2 low no puede ser considerado un subtipo molecular, pero es importante reconocer a estos pacientes dado que en el ensayo clínico DESTINY-Breast04, trastuzumab deruxtecan (T-DXd) demostró que incluso menos receptores HER2 en las células cancerosas pueden ser suficientes para obtener beneficio clínico ^20^^,^^21^. La evaluación de este subgrupo, en especial en aquellos pacientes triple negativo, es vital ya que permitirá terapias específicas, nuevos ensayos clínicos y el desarrollo de técnicas más precisas para evaluar el estado de HER2 ^(^^22^.

Algunas limitaciones encontradas fueron, la carencia de estadística nacional o regional sobre pacientes HER2 low para comparación de resultados, ausencia de datos en las historias clínicas sobre el estado actual de los pacientes dificultando el análisis de sobrevida de gran parte de la muestra, y ausencia de pruebas genéticas que pudieran enriquecer los resultados.

El perfil de subtipificación de cáncer de mama en pacientes diagnosticadas en el Instituto Regional de Enfermedades Neoplásica del Norte durante el 2016 a 2023 difiere de estudios previos, mostrando un incremento notable del cáncer de mama subtipo luminal B a expensas de un decremento del subtipo luminal A. Asimismo, se halló que el subgrupo HER2 low representa la cuarta parte de aquellos pacientes antes considerados sin expresión de estos receptores, abriendo para ellos una oportunidad terapéutica. Esto implica que las políticas de salud pública deben involucrarse y cambiar según el contexto. Contribuir de manera perenne en la investigación, programas de detección precoz más eficaces, adquisición de tratamientos específicos, garantizar recurso humano capacitado, y crear alianzas para la medicina personalizada.
